# Supplementary material for: Human retinal organoids release extracellular vesicles that regulate gene expression in target human retinal progenitor cells
Source: Sci Rep. 2021 Oct 26;11:21128. doi: 10.1038/s41598-021-00542-w (PMC8548301; doi:10.1038/s41598-021-00542-w)
Supplement: Supplementary file 1 — Supplementary Information 1. [file 41598_2021_542_MOESM1_ESM.docx]

**Human retinal organoids release extracellular vesicles that regulate gene expression in target human retinal progenitor cells**

Jing Zhou1,2,#, Miguel Flores-Bellver3#, Jianbo Pan4, Alberto Benito-Martin5, Cui Shi1, Onyekwere Onwumere1, Jason Mighty1,2, Jiang Qian4, Xiufeng Zhong6, Tasmim Hogue1, Baffour Amponsah-Antwi1, Linda Einbond1, Rajendra Gharbaran7, Hao Wu1,2, Bo-Juen Chen8, Zhiliang Zheng1, Tatyana Tchaikovskaya9, Xusheng Zhang10, Hector Peinado11, Maria Valeria Canto-Soler3* and Stephen Redenti1,2,12*

1. Lehman College, 250 Bedford Park Boulevard West, Bronx, NY 10468 USA

2. Biology Doctoral Program, The Graduate School and University Center, City University of New York,

365 5th Avenue, New York, NY 10016 USA

3. CellSight Ocular Stem Cell and Regeneration Program, Department of Ophthalmology, Sue Anschutz- Rodgers Eye Center, University of Colorado, 12800 East 19th Avenue, Aurora, CO 80045 USA

4. Department of Ophthalmology, Johns Hopkins University School of Medicine, Baltimore, MD, 21205, USA.

5. Departments of Pediatrics, Hematology/Oncology Division, Weill Medical College of Cornell University,

413 E. 69th St., New York, NY 10021 USA

6. State Key Laboratory of Ophthalmology, Zhongshan Ophthalmic Center, Sun Yat-sen University, Guangzhou, Guangdong, China

7. Bronx Community College, 2155 University Ave, Bronx, NY 10453, USA.

8. New York Genome Center, New York, NY, 10013, USA.

9. Department of Medicine, Liver Research Center, Albert Einstein College of Medicine, Bronx, New York, United States

10. Department of Medicine, Computational Genomics Core in Department of Genetics, Albert Einstein College of Medicine, Bronx, New York, United States

11. Microenvironment and Metastasis Laboratory, Department of Molecular Oncology, Spanish National

Cancer Research Centre (CNIO), Madrid, E28029, Spain

12. Biochemistry Doctoral Program, The Graduate School, City University of New York, 365 Fifth Avenue, New York, NY 10016 USA

# co-first authors

[* corresponding authors: valeria.canto-soler@](mailto:valeria.canto-soler@cuanschutz.edu)[cuanschutz.edu, stephen.redenti@lehman.cuny.edu](mailto:stephen.redenti@lehman.cuny.edu)

**Supplemental Material**

**Supplemental Video SV1**. **Nanosight live diffraction analysis of EVs released by hiPSC-derived 3D Retinal organoids.** The video shows the brownian motion exhibited by EVs in solution; EVs were collected from the supernatant of D63 hiPSC-derived retinal organoids after 24 hr.

**Supplemental Table ST1A (ST1A also provided as excel file), ST1B, ST1C (ST1B, ST1C also provided as excel files). Total miRNA, piRNA and tRNA present in hiPSC-derived 3D retinal organoids at D42, D63 and D90.**

**Supplemental Table ST1A. Total miRNA identified in 3D retinal organoids**

| hsa-miR-132-3p | RO | RO |  |
| --- | --- | --- | --- |
| hsa-miR-135a-3p | RO | RO |  |
| hsa-miR-135a-5p | RO | RO | RO |
| hsa-miR-135b-5p | RO | RO | RO |
| hsa-miR-139-5p | RO | RO |  |
| hsa-miR-140-5p | RO | RO |  |
| hsa-miR-146b-5p | RO | RO | RO |
| hsa-miR-148a-5p | RO | RO |  |
| hsa-miR-148b-5p | RO | RO |  |
| hsa-miR-149-5p | RO | RO | RO |
| hsa-miR-151a-5p | RO | RO |  |
| hsa-miR-15b-3p | RO | RO |  |
| hsa-miR-15b-5p | RO | RO |  |
| hsa-miR-16-2-3p | RO | RO | RO |
| hsa-miR-16-5p | RO | RO | RO |
| hsa-miR-181a-2-3p | RO | RO | RO |
| hsa-miR-181a-3p | RO | RO |  |
| hsa-miR-181c-3p | RO | RO |  |
| hsa-miR-181d-5p | RO | RO | RO |
| hsa-miR-182-5p | RO | RO | RO |
| hsa-miR-183-5p | RO | RO | RO |
| hsa-miR-185-5p | RO | RO |  |
| hsa-miR-18a-3p | RO | RO |  |
| hsa-miR-18a-5p | RO | RO |  |
| hsa-miR-190b | RO | RO | RO |
| hsa-miR-197-3p | RO | RO | RO |
| **miRNA** | **D42** | **D63** | **D90** |
| hsa-miR-19a-3p | RO | RO |  |
| hsa-miR-19b-3p | RO | RO |  |
| hsa-miR-20b-5p | RO | RO | RO |
| hsa-miR-210-3p | RO | RO | RO |
| hsa-miR-210-5p | RO | RO |  |
| hsa-miR-211-5p | RO | RO |  |
| hsa-miR-2110 | RO | RO |  |
| hsa-miR-216a-3p | RO | RO | RO |
| hsa-miR-216a-5p | RO | RO | RO |
| hsa-miR-216b-3p | RO | RO |  |
| hsa-miR-218-5p | RO | RO | RO |
| hsa-miR-219a-1-3p | RO | RO |  |
| hsa-miR-221-3p | RO | RO | RO |

| **miRNA** | **D42** | **D63** | **D90** |
| --- | --- | --- | --- |
| hsa-miR-4488 | RO | RO | RO |
| hsa-miR-4497 | RO | RO |  |
| hsa-miR-1298-5p | RO | RO |  |
| hsa-miR-184 | RO | RO |  |
| hsa-miR-204-3p | RO | RO |  |
| hsa-miR-4301 | RO | RO |  |
| hsa-miR-99b-3p | RO | RO | RO |
| hsa-miR-1307-3p | RO | RO | RO |
| hsa-miR-7704 | RO | RO |  |
| hsa-miR-92b-3p | RO | RO |  |
| hsa-miR-92b-5p | RO | RO |  |
| hsa-miR-95-3p | RO | RO | RO |
| hsa-miR-105-5p | RO | RO |  |
| hsa-miR-106a-5p | RO | RO |  |
| hsa-miR-106b-3p | RO | RO |  |
| hsa-miR-106b-5p | RO | RO |  |
| hsa-miR-107 | RO | RO |  |
| hsa-miR-1180-3p | RO | RO | RO |
| hsa-miR-1226-3p | RO | RO |  |
| hsa-miR-124-3p | RO | RO | RO |
| hsa-miR-124-5p | RO | RO |  |
| hsa-miR-1247-3p | RO | RO |  |
| hsa-miR-1247-5p | RO | RO | RO |
| hsa-miR-1248 | RO | RO | RO |
| hsa-miR-125b-1-3p | RO | RO |  |
| hsa-miR-125b-2-3p | RO | RO |  |
| hsa-miR-1260a | RO | RO | RO |
| hsa-miR-1260b | RO | RO | RO |
| hsa-miR-1261 | RO | RO |  |
| hsa-miR-1271-5p | RO | RO |  |
| hsa-miR-1275 | RO | RO |  |
| hsa-miR-1287-5p | RO | RO |  |
| hsa-miR-129-2-3p | RO |  |  |
| hsa-miR-129-5p | RO | RO |  |
| hsa-miR-1301-3p | RO | RO |  |
| hsa-miR-1306-5p | RO | RO |  |
| hsa-miR-130a-3p | RO | RO |  |
| hsa-miR-130b-3p | RO | RO |  |
| hsa-miR-130b-5p | RO | RO |  |

| hsa-miR-363-5p | RO | RO |  |
| --- | --- | --- | --- |
| hsa-miR-374a-3p | RO | RO |  |
| hsa-miR-374a-5p | RO | RO |  |
| hsa-miR-374b-5p | RO | RO | RO |
| hsa-miR-383-5p | RO | RO |  |
| hsa-miR-421 | RO | RO |  |
| hsa-miR-425-3p | RO | RO |  |
| hsa-miR-425-5p | RO | RO |  |
| hsa-miR-4443 | RO | RO |  |
| hsa-miR-4455 | RO | RO |  |
| hsa-miR-4485 | RO | RO |  |
| hsa-miR-449c-5p | RO | RO |  |
| hsa-miR-452-5p | RO | RO | RO |
| hsa-miR-454-3p | RO | RO |  |
| hsa-miR-454-5p | RO | RO |  |
| hsa-miR-455-3p | RO | RO |  |
| hsa-miR-455-5p | RO | RO |  |
| hsa-miR-4791 | RO | RO |  |
| hsa-miR-483-3p | RO | RO |  |
| hsa-miR-484 | RO | RO |  |
| hsa-miR-499a-5p | RO | RO |  |
| hsa-miR-500a-3p | RO | RO |  |
| hsa-miR-501-3p | RO | RO |  |
| hsa-miR-501-5p | RO | RO |  |
| hsa-miR-5010-3p | RO | RO |  |
| hsa-miR-504-5p | RO | RO |  |
| hsa-miR-505-3p | RO | RO |  |
| **miRNA** | **D42** | **D63** | **D90** |
| hsa-miR-532-3p | RO | RO |  |
| hsa-miR-548ah-3p | RO | RO |  |
| hsa-miR-548p | RO | RO |  |
| hsa-miR-551b-5p | RO | RO |  |
| hsa-miR-5701 | RO | RO | RO |
| hsa-miR-574-3p | RO | RO |  |
| hsa-miR-582-3p | RO | RO |  |
| hsa-miR-589-5p | RO | RO |  |
| hsa-miR-598-3p | RO | RO |  |
| hsa-miR-619-5p | RO | RO |  |
| hsa-miR-625-3p | RO | RO |  |
| hsa-miR-629-5p | RO | RO | RO |
| hsa-miR-651-5p | RO | RO |  |

| hsa-miR-221-5p | RO | RO |  |
| --- | --- | --- | --- |
| hsa-miR-222-3p | RO | RO | RO |
| hsa-miR-23a-3p | RO | RO |  |
| hsa-miR-23b-3p | RO | RO | RO |
| hsa-miR-25-5p | RO | RO |  |
| hsa-miR-28-3p | RO | RO |  |
| hsa-miR-28-5p | RO | RO |  |
| hsa-miR-296-3p | RO | RO |  |
| hsa-miR-301a-5p | RO | RO |  |
| hsa-miR-301b | RO | RO |  |
| hsa-miR-3065-5p | RO | RO |  |
| hsa-miR-30a-3p | RO | RO | RO |
| hsa-miR-30b-5p | RO | RO | RO |
| hsa-miR-30c-2-3p | RO | RO |  |
| hsa-miR-30e-3p | RO | RO | RO |
| hsa-miR-3177-3p | RO | RO |  |
| hsa-miR-3178 | RO | RO |  |
| hsa-miR-3195 | RO | RO | RO |
| hsa-miR-3196 | RO | RO |  |
| hsa-miR-32-3p | RO | RO |  |
| hsa-miR-32-5p | RO | RO |  |
| hsa-miR-324-3p | RO | RO |  |
| hsa-miR-328-3p | RO | RO |  |
| hsa-miR-330-3p | RO | RO |  |
| hsa-miR-331-3p | RO | RO |  |
| hsa-miR-335-3p | RO | RO |  |
| hsa-miR-335-5p | RO | RO |  |
| hsa-miR-338-5p | RO | RO | RO |
| hsa-miR-339-3p | RO | RO |  |
| hsa-miR-339-5p | RO | RO |  |
| hsa-miR-340-3p | RO | RO |  |
| hsa-miR-342-3p | RO | RO | RO |
| hsa-miR-342-5p | RO | RO |  |
| hsa-miR-345-5p | RO | RO |  |
| hsa-miR-34a-5p | RO | RO |  |
| hsa-miR-34c-5p | RO | RO | RO |
| hsa-miR-3607-3p | RO | RO | RO |
| hsa-miR-361-3p | RO | RO |  |
| hsa-miR-361-5p | RO | RO |  |
| hsa-miR-3615 | RO | RO |  |
| hsa-miR-362-5p | RO | RO |  |

| hsa-let-7d-5p | RO | RO |  |
| --- | --- | --- | --- |
| hsa-miR-1 | RO | RO |  |
| hsa-miR-17-5p | RO | RO |  |
| hsa-miR-181b-5p | RO | RO |  |
| hsa-miR-204-5p | RO | RO |  |
| hsa-miR-20a-5p | RO | RO |  |
| hsa-miR-224-5p | RO | RO |  |
| hsa-miR-30c-5p | RO | RO |  |
| hsa-miR-30e-5p | RO | RO |  |
| hsa-miR-363-3p | RO | RO |  |
| hsa-miR-744-5p | RO | RO |  |
| hsa-miR-191-5p | RO | RORO |  |
| hsa-miR-25-3p | RO | RO |  |
| hsa-miR-320c | RO | RO |  |
| hsa-miR-340-5p | RO | RO |  |
| hsa-miR-574-5p | RO | RO |  |
| hsa-miR-93-5p | RO | RO |  |
| hsa-miR-424-3p |  |  | RO |
| hsa-miR-424-5p |  |  | RO |
| hsa-miR-450a-5p |  |  | RO |
| hsa-miR-503-5p |  |  | RO |
| hsa-miR-542-3p |  |  | RO |

| hsa-miR-652-3p | RO | RO | RO |
| --- | --- | --- | --- |
| hsa-miR-660-5p | RO | RO |  |
| hsa-miR-671-3p | RO | RO |  |
| hsa-miR-708-3p | RO | RO |  |
| hsa-miR-708-5p | RO | RO | RO |
| hsa-miR-760 | RO | RO |  |
| hsa-miR-7641 | RO | RO | RO |
| hsa-miR-766-3p | RO | RO |  |
| hsa-miR-767-5p | RO | RO |  |
| hsa-miR-769-5p | RO | RO | RO |
| hsa-miR-7706 | RO | RO |  |
| hsa-miR-7977 | RO | RO |  |
| hsa-miR-874-3p | RO | RO |  |
| hsa-miR-877-3p | RO | RO |  |
| hsa-miR-877-5p | RO | RO |  |
| hsa-miR-9-3p | RO | RO | RO |
| hsa-miR-92a-1-5p | RO | RO |  |
| hsa-miR-93-3p | RO | RO |  |
| hsa-miR-935 | RO | RO |  |
| hsa-miR-941 | RO | RO | RO |
| hsa-miR-216b-5p | RO | RO |  |
| hsa-miR-486-5p | RO | RO |  |


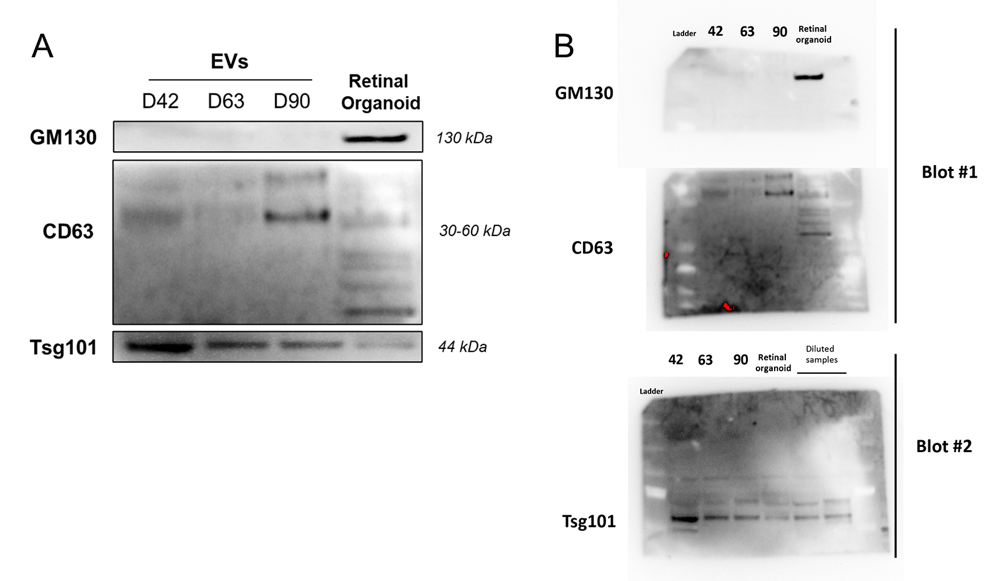


**Supplemental Figure 1 (SF1). Western blot confirmation of EV markers**. A) Combined immunoblots against well-known EV specific markers (TSG-101 and CD63) and an intracellular Golgi membrane protein (GM130) confirmed the purity of EV preparations obtained from human Retinal Organoids. B) Original unprocessed full length blots (#1,#2).


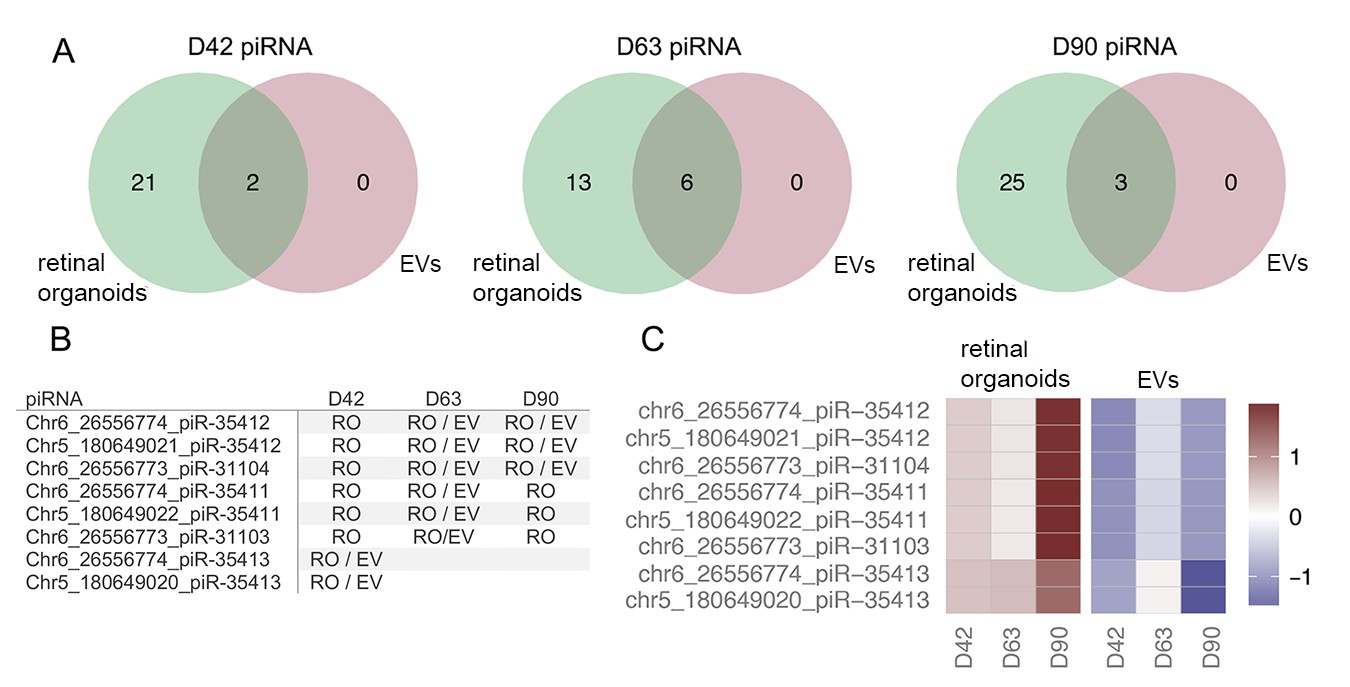


**Supplemental Figure 2 (SF2). Next generation sequencing analysis of piRNA expression in hiPSC- derived 3D retinal organoids and EVs.** A) The Venn diagrams show the number of piRNAs expressed

>5 RPM in 3D retinal organoids, EVs, or both samples. All piRNA species detected were shared between

3D retinal organoids and EVs at D42 (n=2), D63 (n=6), and D90 (n=3) with no piRNAs unique to EVs. 3D retina organoid samples at D42, D63 and D90 had 21, 13, and 25 unique piRNAs with >5 RPM, respectively. D42, D63, and D90 represent the time (in days) when the samples were collected. B) piRNA species that were expressed >5 RPM in EVs (EV), retinal organoids (RO), or both (RO/EV) at any time point. piRNAs without annotation were either detected below the threshold or detected also in controls. The combinations of piRNA species present in EVs were unique between each time point and the relative expression level of each of these piRNAs was different between retina and EVs at all time points analyzed. C) Heatmap of piRNA expression levels in retinal organoids and EVs; piRNA species expressed >5 RPM in EVs at any time points are shown. The RPM values were log-transformed and standardized across samples for visualization. For each RNA, red-colored samples represent higher expression values than do blue colored samples. The color bar shows the range of standardized expression values.


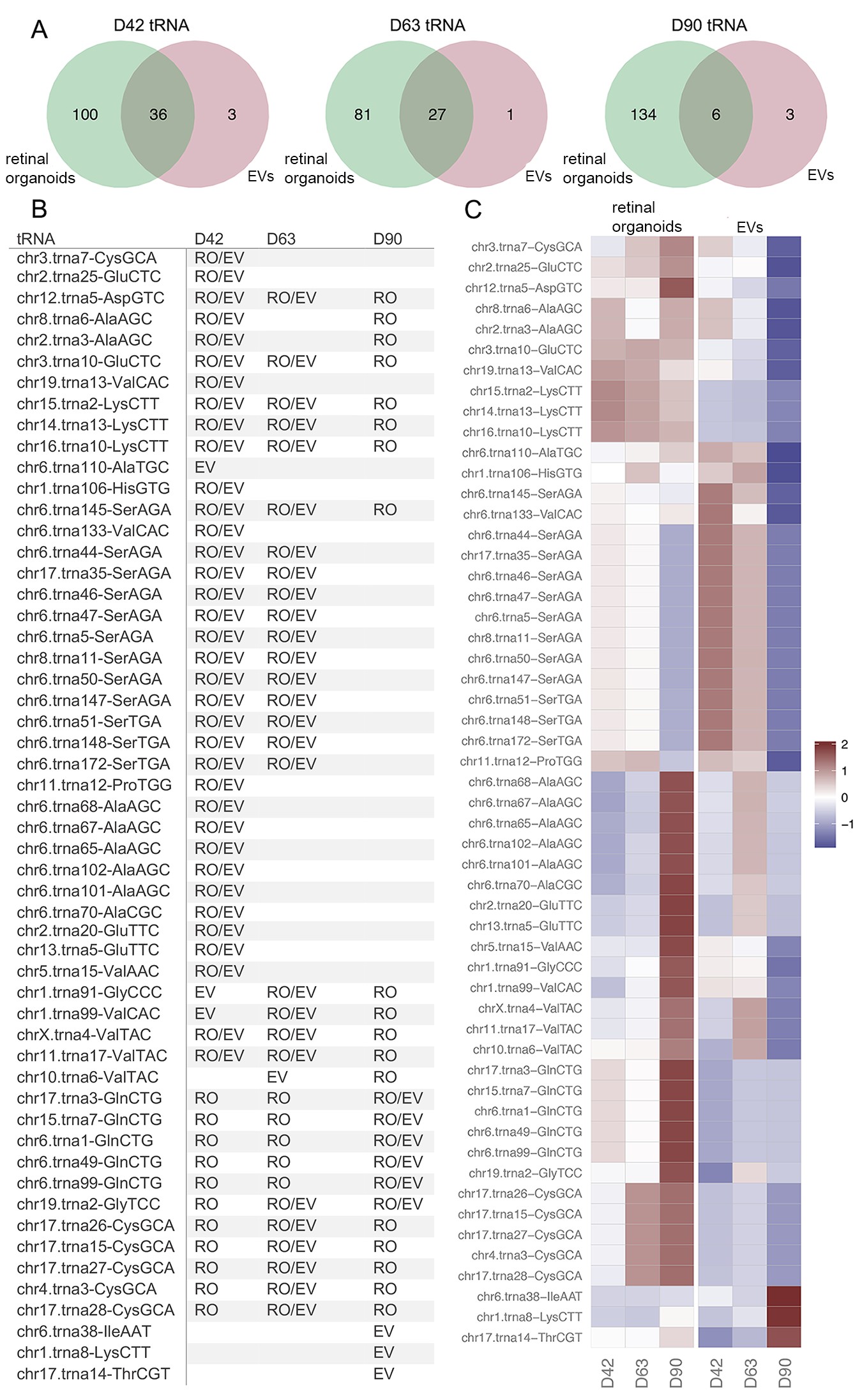


**Supplemental Figure 3 (SF3). Next generation sequencing analysis of tRNA expression in hiPSC- derived 3D retinal organoids and EVs.** Compared with miRNA and piRNA species, tRNA exhibited the highest number of species contained within EVs released from developing hiPSC-derived 3D retinal organoids. A) The Venn diagrams show the number of tRNAs expressed >5 RPM in 3D retina, EVs, or both samples. D42, D63, and D90 represent the time (in days) when the samples were collected. B) Selected tRNAs (23 out of 54) that were expressed >5 RPM in EVs (EV), retinal organoids (RO), or both RO/EV) at any time point. tRNAs without annotation were either detected below the threshold or detected also in controls. Interestingly, tRNA species exclusively detected in EVs were unique at each time point, and the relative expression level of each of these tRNAs was different between retinal organoids and EVs at all time points analyzed as shown in C. C) Heatmap of tRNA levels in retinal organoids and EVs; tRNAs that were expressed >5 RPM in EVs at any time point are shown. D42 EVs contained higher expression levels of tRNA >5 RPM compared to D63 and D90. The RPM values were log-transformed

and standardized across samples for visualization. For each RNA, red-colored samples represent higher expression values than do blue-colored samples.

**Supplemental Table 1B. Total piRNA identified in 3D retinal organoids.**

| **piRNA** | **D42** | **D63** | **D90** |
| --- | --- | --- | --- |
| chr5_180649021_piR-35412 | RO | RO | RO |
| chr6_26554350_piR-36037 |  | RO |  |
| chr6_26556773_piR-31104 | RO | RO | RO |
| chr6_26556774_piR-35412 | RO | RO | RO |
| chr5_180649020_piR-35413 | RO |  |  |
| chr6_26556774_piR-35413 | RO |  |  |
| chr5_180649022_piR-35411 | RO | RO | RO |
| chr6_26556773_piR-31103 | RO | RO | RO |
| chr6_26556774_piR-35411 | RO |  | RO |
| chr5_180649019_piR-33468 | RO | RO | RO |
| chr6_26556777_piR-33468 | RO | RO | RO |
| chr22_39709882_piR-36716 | RO | RO | RO |
| chr22_39709883_piR-36715 | RO | RO | RO |
| chr22_39709884_piR-36714 | RO | RO | RO |
| chr22_39709885_piR-36713 | RO | RO | RO |
| chr5_180645310_piR-43993 |  |  | RO |
| chr5_180645311_piR-60576 |  |  | RO |
| chr5_180649001_piR-39018 |  |  | RO |
| chr5_180649021_piR-33161 | RO | RO | RO |
| chr5_180649023_piR-35410 | RO | RO | RO |
| chr5_180649433_piR-43994 |  |  | RO |
| chr5_180649434_piR-60577 | RO |  | RO |
| chr5_180649435_piR-43993 |  |  | RO |
| chr5_180649436_piR-60576 |  |  | RO |
| chr6_26538284_piR-60577 | RO |  | RO |
| chr6_26538285_piR-43993 |  |  | RO |
| chr6_26538285 piR-43994 |  |  | RO |
| chr6_26556774_piR-35410 | RO | RO | RO |
| chr6_26556776_piR-33161 | RO | RO | RO |
| chr6_26556793_piR-39018 |  |  | RO |
| chr8_56986429_piR-55152 | RO | RO |  |
| chr8_56986430_piR-55151 | RO | RO |  |
| chr8_56986432_piR-55150 | RO |  |  |

**Supplemental Table 1C. Total tRNA identified in 3D retinal organoids**.

| chr1.trna15-GlnCTG | RO | RO |  |
| --- | --- | --- | --- |
| chr1.trna19-GlnCTG | RO | RO |  |
| chr1.trna28-GlnCTG | RO |  |  |
| chr1.trna32-MetCAT | RO | RO |  |
| chr1.trna45-GlyTCC | RO | RO |  |
| chr1.trna56-ThrTGT | RO | RO | RO |
| chr1.trna58-LeuCAA | RO | RO | RO |
| chr1.trna67-LeuCAG | RO |  | RO |
| chr1.trna69-AspGTC | RO | RO | RO |
| chr1.trna70-GlyTCC | RO | RO | RO |
| chr1.trna72-AspGTC | RO | RO | RO |
| chr1.trna73-GlyTCC | RO | RO |  |
| chr1.trna75-AspGTC | RO | RO |  |
| chr1.trna76-GlyTCC | RO | RO |  |
| chr1.trna78-AspGTC | RO | RO |  |
| chr1.trna79-GlyTCC | RO | RO | RO |
| chr1.trna81-AspGTC | RO | RO | RO |
| chr1.trna82-GlyTCC | RO | RO |  |
| chr11.trna16-ValTAC | RO |  |  |
| chr11.trna8-SerGCT | RO |  |  |
| chr12.trna10-AspGTC | RO | RO | RO |
| chr12.trna12-AspGTC | RO | RO |  |
| chr12.trna4-AspGTC | RO | RO | RO |
| chr12.trna6-TrpCCA | RO |  |  |
| chr12.trna8-AlaTGC | RO | RO |  |
| chr14.trna1-LeuAAG | RO | RO | RO |
| chr14.trna2-LeuTAG | RO |  | RO |
| chr14.trna20-ThrTGT | RO | RO | RO |
| chr14.trna21-ThrTGT | RO | RO | RO |
| chr14.trna4-ThrTGT | RO | RO |  |
| chr15.trna6-LysCTT | RO | RO | RO |
| chr16.trna12-ArgCCT | RO | RO |  |
| chr16.trna16-LeuAAG | RO | RO | RO |
| chr16.trna18-GlyGCC | RO | RO | RO |
| chr16.trna2-ArgCCT | RO | RO |  |
| chr16.trna25-GlyGCC | RO | RO | RO |
| chr16.trna7-LysCTT | RO | RO |  |
| chr17.trna10-GlyTCC | RO | RO |  |
| chr17.trna18-ArgCCT | RO | RO | RO |
| chr17.trna20-MetCAT | RO | RO |  |
| chr17.trna21-ArgCCT | RO | RO |  |
| chr17.trna23-ArgCCG | RO | RO |  |
| chr17.trna38-AspGTC | RO | RO | RO |
| chr17.trna42-LeuTAG | RO | RO | RO |
| Chr17.random.trna1-ArgCCG | RO | RO | RO |
| chr18.trna4-LysCTT | RO | RO |  |
| chr19.trna10-IleTAT | RO | RO |  |
| chr19.trna6-LysCTT | RO | RO |  |
| chr2.trna2-TyrGTA | RO | RO | RO |
| chr3.trna7-CysGCA | RO |  |  |
| **tRNA** | **D42** | **D63** | **D90** |
| chr5.trna11-LysCTT | RO | RO | RO |

| **tRNA** | **D42** | **D63** | **D90** |
| --- | --- | --- | --- |
| chr11.trna17-ValTAC | RO | RO | RO |
| chr19.trna2-GlyTCC | RO | RO | RO |
| chrX.trna4-ValTAC | RO | RO | RO |
| chr12.trna5-AspGTC | RO | RO | RO |
| chr14.trna13-LysCTT | RO | RO | RO |
| chr15.trna2-LysCTT | RO | RO | RO |
| chr16.trna10-LysCTT | RO | RO | RO |
| chr17.trna35-SerAGA | RO | RO | RO |
| chr2.trna25-GluCTC | RO |  | RO |
| chr3.trna10-GluCTC | RO | RO | RO |
| chr6.trna145-SerAGA | RO | RO | RO |
| chr6.trna147-SerAGA | RO | RO | RO |
| chr6.trna147-SerAGA | RO | RO | RO |
| chr6.trna147-SerAGA | RO | RO | RO |
| chr6.trna147-SerAGA | RO | RO | RO |
| chr6.trna147-SerAGA | RO | RO | RO |
| chr6.trna147-SerAGA | RO | RO | RO |
| chr6.trna147-SerAGA | RO | RO | RO |
| chr6.trna147-SerAGA | RO | RO | RO |
| chr6.trna147-SerAGA | RO | RO | RO |
| chr6.trna147-SerAGA | RO | RO | RO |
| chr6.trna147-SerAGA | RO | RO | RO |
| chr17.trna15-CysGCA | RO | RO | RO |
| chr17.trna26-CysGCA | RO | RO | RO |
| chr17.trna27-CysGCA | RO | RO | RO |
| chr17.trna28-CysGCA | RO | RO |  |
| chr17.trna3-GlnCTG | RO | RO | RO |
| chr2.trna3-AlaAGC | RO |  |  |
| chr4.trna3-CysGCA | RO | RO |  |
| chr5.trna15-ValAAC | RO |  | RO |
| chr6.trna1-GlnCTG | RO | RO | RO |
| chr6.trna49-GlnCTG | RO | RO | RO |
| chr6.trna99-GlnCTG | RO | RO | RO |
| chr8.trna6-AlaAGC | RO |  |  |
| chr11.trna12-ProTGG | RO |  |  |
| chr13.trna5-GluTTC | RO |  | RO |
| chr2.trna20-GluTTC | RO |  | RO |
| chr6.trna101-AlaAGC | RO |  |  |
| chr6.trna102-AlaAGC | RO |  | RO |
| chr6.trna146-GlnCTG | RO | RO |  |
| chr6.trna42-GlnCTG | RO | RO | RO |
| chr6.trna65-AlaAGC | RO |  | RO |
| chr6.trna67-AlaAGC | RO |  | RO |
| chr6.trna68-AlaAGC | RO |  | RO |
| chr6.trna70-AlaCGC | RO |  | RO |
| chr1.trna106-HisGTG | RO |  | RO |
| chr1.trna112-GlnCTG | RO |  | RO |
| chr1.trna117-GlyTCC | RO | RO | RO |
| chr1.trna119-LysCTT | RO | RO | RO |
| chr1.trna127-LysCTT | RO |  | RO |
| chr1.trna128-LysCTT | RO |  | RO |

| cchr1.trna26-AsnGTT |  |  | RO |
| --- | --- | --- | --- |
| cchr1.trna43-GlyGCC |  |  | RO |
| chr1.trna50-AsnGTT |  |  | RO |
| chr1.trna83-AsnGTT |  |  | RO |
| chr1.trna88-ArgCCT |  |  | RO |
| chr1.trna96-ArgCCT |  |  | RO |
| chr10.trna4-AsnGTT |  |  | RO |
| cchr10.trna6-ValTAC |  |  | RO |
| cchr13.trna2-GluCTC |  |  | RO |
| cchr13.trna7-AsnGTT |  |  | RO |
| cchr14.trna16-TyrGTA |  |  | RO |
| cchr14.trna19-TyrGTA |  |  | RO |
| cchr14.trna5-TyrGTA |  |  | RO |
| chr15.trna10-SerGCT |  |  | RO |
| chr16.trna1-ArgCCG |  |  | RO |
| chr16.trna20-MetCAT |  |  | RO |
| chr17.trna12-TrpCCA |  |  | RO |
| chr17.trna16-GlnTTG |  |  | RO |
| chr17.trna31-AsnGTT |  |  | RO |
| cchr17.trna7-SerGCT |  |  | RO |
| cchr19.trna1-AsnGTT |  |  | RO |
| chr19.trna5-LysCTT |  |  | RO |
| chr2.trna17-GluTTC |  |  | RO |
| chr2.trna5-IleTAT |  |  | RO |
| chr3.trna11-ArgACG |  |  | RO |
| chr3.trna4-HisGTG |  |  | RO |
| chr6.trna108-AlaAGC |  |  | RO |
| chr6.trna114-ArgCCG |  |  | RO |
| chr6.trna123-SerGCT |  |  | RO |
| chr6.trna130-GlnTTG |  |  | RO |
| chr6.trna138-ArgACG |  |  | RO |
| chr6.trna156-ArgACG |  |  | RO |
| chr6.trna162-MetCAT |  |  | RO |
| chr6.trna164-MetCAT |  |  | RO |
| chr6.trna166-AlaAGC |  |  | RO |
| chr6.trna173-GlnTTG |  |  | RO |
| chr6.trna174-GlnTTG |  |  | RO |
| chr6.trna175-SerGCT |  |  | RO |
| chr6.trna27-MetCAT |  |  | RO |
| chr6.trna31-SerGCT |  |  | RO |
| chr6.trna36-ArgACG |  |  | RO |
| chr6.trna43-SerGCT |  |  | RO |
| chr6.trna62-SerGCT |  |  | RO |
| chr6.trna73-ArgCCG |  |  | RO |
| chr6.trna75-MetCAT |  |  | RO |
| chr6.trna97-MetCAT |  |  | RO |

| chr5.trna16-LeuAAG | RO |  |  |
| --- | --- | --- | --- |
| chr5.trna19-LeuAAG | RO |  | RO |
| chr5.trna22-AspGTC | RO |  | RO |
| chr5.trna3-LeuAAG | RO |  | RO |
| chr5.trna7-LeuAAG | RO | RO | RO |
| chr5.trna9-LysCTT | RO | RO | RO |
| chr6.trna100-LeuCAA | RO | RO | RO |
| chr6.trna126-LeuAAG | RO | RO | RO |
| chr6.trna129-MetCAT | RO | RO |  |
| chr6.trna13-LysCTT | RO | RO |  |
| chr6.trna131-GlnCTG | RO |  |  |
| chr6.trna133-ValCAC | RO |  |  |
| chr6.trna14-TyrGTA | RO |  | RO |
| chr6.trna140-LeuCAA | RO |  | RO |
| chr6.trna142-MetCAT | RO | RO |  |
| chr6.trna143-LysTTT | RO | RO |  |
| chr6.trna144-AspGTC | RO | RO |  |
| chr6.trna149-LysTTT | RO | RO | RO |
| chr6.trna150-MetCAT | RO | RO |  |
| chr6.trna151-ThrCGT | RO | RO |  |
| chr6.trna169-MetCAT | RO | RO |  |
| chr6.trna171-MetCAT | RO | RO |  |
| chr6.trna2-MetCAT | RO | RO | RO |
| chr6.trna45-AspGTC | RO | RO | RO |
| chr6.trna48-AspGTC | RO | RO | RO |
| chr6.trna61-MetCAT | RO | RO |  |
| chr6.trna74-LeuCAA | RO | RO |  |
| chr6.trna78-LeuAAG | RO | RO | RO |
| chr6.trna83-LeuTAA | RO | RO |  |
| chr7.trna3-ArgCCT | RO | RO | RO |
| chr9.trna6-AspGTC | RO |  |  |
| chr16.trna34-GlyCCC | RO |  | RO |
| chr19.trna13-ValCAC | RO |  | RO |
| chr2.trna27-GlyCCC | RO |  | RO |
| chr1.trna91-GlyCCC |  | RO | RO |
| chr1.trna99-ValCAC |  | RO | RO |
| chr19.trna8-SeC(e)TCA |  | RO | RO |
| chr5.trna1-CysACA |  | RO |  |
| chr6.trna98-LeuAAG |  | RO |  |
| chr7.trna21-CysGCA |  | RO |  |
| chr7.trna5-CysGCA |  | RO |  |
| chr16.trna27-LeuTAG |  | RO |  |
| chr1.trna86-ArgTCT |  |  | RO |
| chr1.trna107-AsnGTT |  |  | RO |
| chr1.trna108-AsnGTT |  |  | RO |
| cchr1.trna22-GlnCTG |  |  | RO |

**Supplemental Video SV2. Live-Cell Microscopy of hiPSC-derived 3D Retinal organoid released EVs internalized by multipotent hRPCs.** Live-cell fluorescence microscopy was performed of labeled hiPSC-derived 3D retinal organoid EVs internalized by hRPCs. Images were acquired in cultured hRPCs with EVs co-labeled with PKH26 (TRITC) and SytoRNA (FITC) overlapping and orange in the video. The nucleus is labeled with Nuncblu (DAPI). Images were acquired every 20 seconds for 10 minutes to visualize intracellular hRPC transport of co-labeled EVs on a 37C incubated stage using a S Fluor 40x Oil objective and Nikon Elements software.


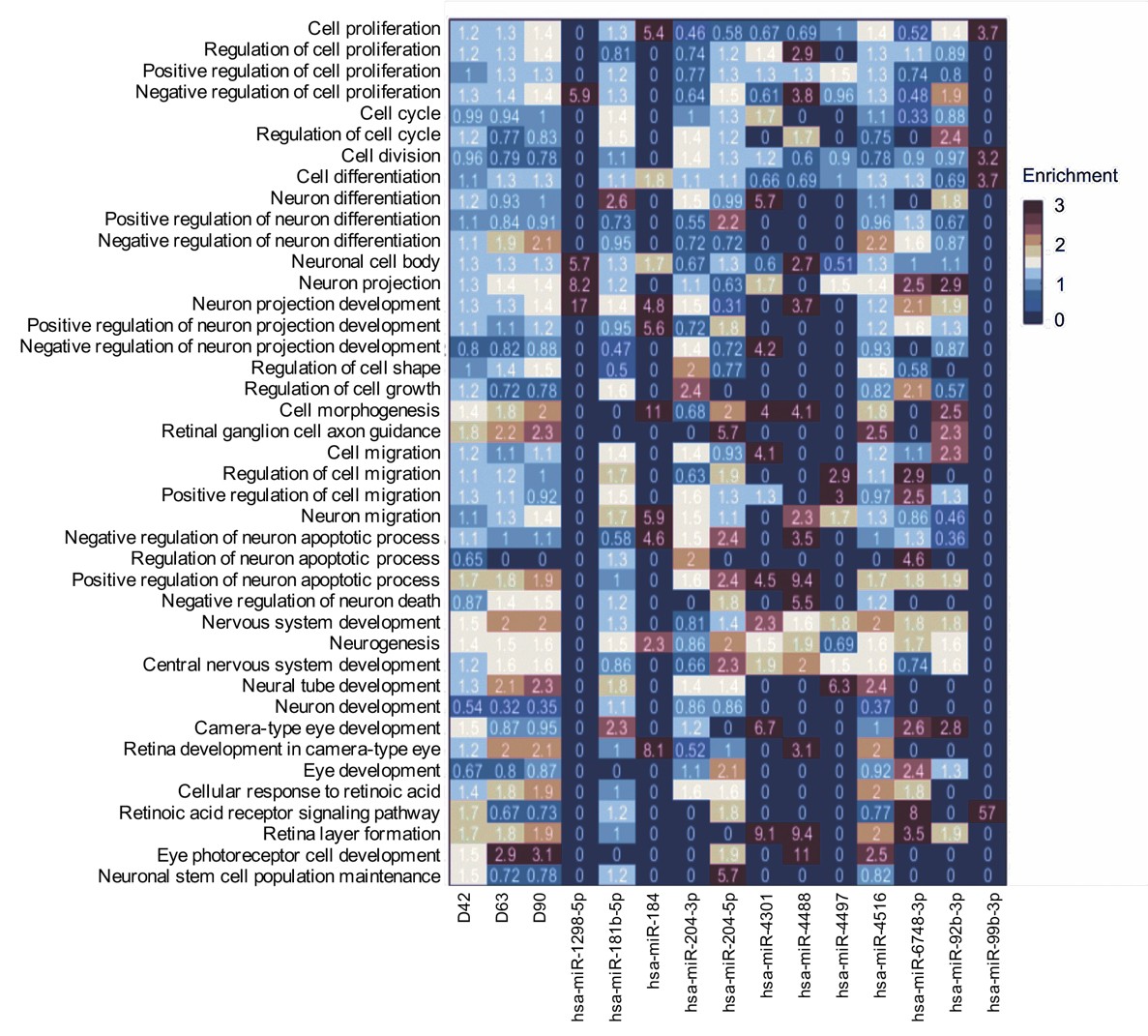


**Supplemental Figure 4 (SF4). Enrichment analysis of Gene Ontology Biological Processes (GOBP) for the predicted targetome of miRNA cargo from EVs released by hiPSC-derived 3D retinal organoids**. The enrichment heatmap reveals biological functions correlated with predicted EV miRNA targets in retinal organoids.

| **Mechanism** | **GO Term** | **Gene**  **Target** | **miRNA** | **D42** | **D63** | **D90** |
| --- | --- | --- | --- | --- | --- | --- |
| Photoreceptor development and differentiation | GO:0001754 eye photoreceptor cell  differentiation | PPP2R3A | hsa-miR-181b-5p | Y |  |  |
|  |  | STAT3 | hsa-miR-4516 | Y | Y | Y |
|  | GO:0042462 eye photoreceptor cell  development | PRKCI | hsa-miR-204-5p | Y |  |  |
|  |  | GNAT1 | hsa-miR-4516 | Y | Y | Y |
|  |  | TULP1 | hsa-miR-4488 | Y | Y | Y |
|  |  | CRB1 | hsa-miR-4516 | Y | Y | Y |
|  |  | RORB | hsa-miR-4516 | Y | Y | Y |
|  | GO:0046533 negative regulation of  photoreceptor cell differentiation | SOX8 | hsa-miR-4516 | Y | Y | Y |
|  | GO:0046549 retinal cone cell development | HCN1 | hsa-miR-181b-5p | Y |  |  |
|  |  | HCN1 | hsa-miR-204-5p | Y |  |  |
|  |  | RORB | hsa-miR-4516 | Y | Y | Y |
|  |  | THY1 | hsa-miR-4516 | Y | Y | Y |
|  | GO:0001917 photoreceptor inner segment | GUCA1B | hsa-miR-204-5p | Y |  |  |
|  |  | DNM3 | hsa-miR-204-3p | Y |  |  |
|  |  | PHLPP2 | hsa-miR-92b-3p | Y |  |  |
|  |  | GNB1 | hsa-miR-184 | Y |  |  |
|  |  | GNB5 | hsa-miR-6748-3p | Y |  |  |
|  |  | DNM2 | hsa-miR-204-5p | Y |  |  |
|  |  | RHO | hsa-miR-4516 | Y | Y | Y |
|  |  | GNAT1 | hsa-miR-4516 | Y | Y | Y |
|  |  | TULP1 | hsa-miR-4488 | Y | Y | Y |
|  |  | DNM3 | hsa-miR-4516 | Y | Y | Y |
|  |  | PHLPP2 | hsa-miR-4516 | Y | Y | Y |
|  | GO:0001750 photoreceptor outer segment | MYO5A | hsa-miR-92b-3p | Y |  |  |
|  |  | PTPRK | hsa-miR-92b-3p | Y |  |  |
|  |  | GNAQ | hsa-miR-92b-3p | Y |  |  |
|  |  | MAP1B | hsa-miR-181b-5p | Y |  |  |
|  |  | MAP1B | hsa-miR-92b-3p | Y |  |  |
|  |  | GNB5 | hsa-miR-6748-3p | Y |  |  |
|  |  | PCDH15 | hsa-miR-204-3p | Y |  |  |
|  |  | PCDH15 | hsa-miR-4301 | Y |  |  |
|  |  | CNGB1 | hsa-miR-204-3p | Y |  |  |
|  |  | RGS9BP | hsa-miR-4497 | Y | Y |  |
|  |  | RHO | hsa-miR-4516 | Y | Y | Y |
|  |  | GNAT1 | hsa-miR-4516 | Y | Y | Y |
|  |  | TULP1 | hsa-miR-4488 | Y | Y | Y |
|  |  | OPN1LW | hsa-miR-4516 | Y | Y | Y |
|  |  | OPN1MW | hsa-miR-4516 | Y | Y | Y |
|  |  | PHLPP2 | hsa-miR-92b-3p | Y |  |  |
|  | GO:0042622 photoreceptor outer segment membrane | GNB1 | hsa-miR-184 | Y |  |  |
|  |  | OPN1MW | hsa-miR-4516 | Y | Y | Y |
|  |  | RHO | hsa-miR-4516 | Y | Y | Y |
|  |  | GNAT1 | hsa-miR-4516 | Y | Y | Y |
|  |  | PHLPP2 | hsa-miR-4516 | Y | Y | Y |
|  |  | NAPEPLD | hsa-miR-4516 | Y | Y | Y |
|  |  | OPN1LW | hsa-miR-4516 | Y | Y | Y |
|  |  | PCDH15 | hsa-miR-204-3p | Y |  |  |
|  | GO:0045494 photoreceptor cell maintenance | PCDH15 | hsa-miR-4301 | Y |  |  |
|  |  | CNGB1 | hsa-miR-204-3p | Y |  |  |
|  |  | CDH23 | hsa-miR-181b-5p | Y |  |  |
|  |  | TULP1 | hsa-miR-4488 | Y | Y | Y |
|  |  | TUB | hsa-miR-4516 | Y | Y | Y |
|  |  | RHO | hsa-miR-4516 | Y | Y | Y |
|  |  | OPN1LW | hsa-miR-4516 | Y | Y | Y |
|  | GO:0009881 photoreceptor activity | OPN1MW | hsa-miR-4516 | Y | Y | Y |
|  |  | RHO | hsa-miR-4516 | Y | Y | Y |
|  |  | OPN1LW | hsa-miR-4516 | Y | Y | Y |
|  |  | OPN1MW | hsa-miR-4516 | Y | Y | Y |
|  |  | RHO | hsa-miR-4516 | Y | Y | Y |
|  |  | ALCAM | hsa-miR-204-5p | Y |  |  |
| Ganglion cell  development and differentiation | GO:0031290 retinal ganglion cell axon guidance | EPHB2 | hsa-miR-204-5p | Y |  |  |
|  |  | ROBO2 | hsa-miR-204-5p | Y |  |  |
|  |  | ROBO2 | hsa-miR-92b-3p | Y |  |  |
|  |  | EFNA5 | hsa-miR-4516 | Y | Y | Y |
|  |  | EPHB3 | hsa-miR-4516 | Y | Y | Y |
|  |  | SEMA4F | hsa-miR-4516 | Y | Y | Y |

**Supplemental Table ST2. hiPSC-derived 3D retinal organoid EV miRNA target genes associated with photoreceptor and ganglion cell differentiation and function**. The table indicates the temporal pattern expression of EV miRNAs (D42, D63, D90) and the predicted target genes with the corresponding GO categories. The analysis shows that hiPSC-derived 3D retinal organoid EV miRNAs target genes associated with ganglion and photoreceptor cell development. Genes were identified within the pool of targeted genes that were associated with mechanisms related to ganglion cell and photoreceptor differentiation and physiology.


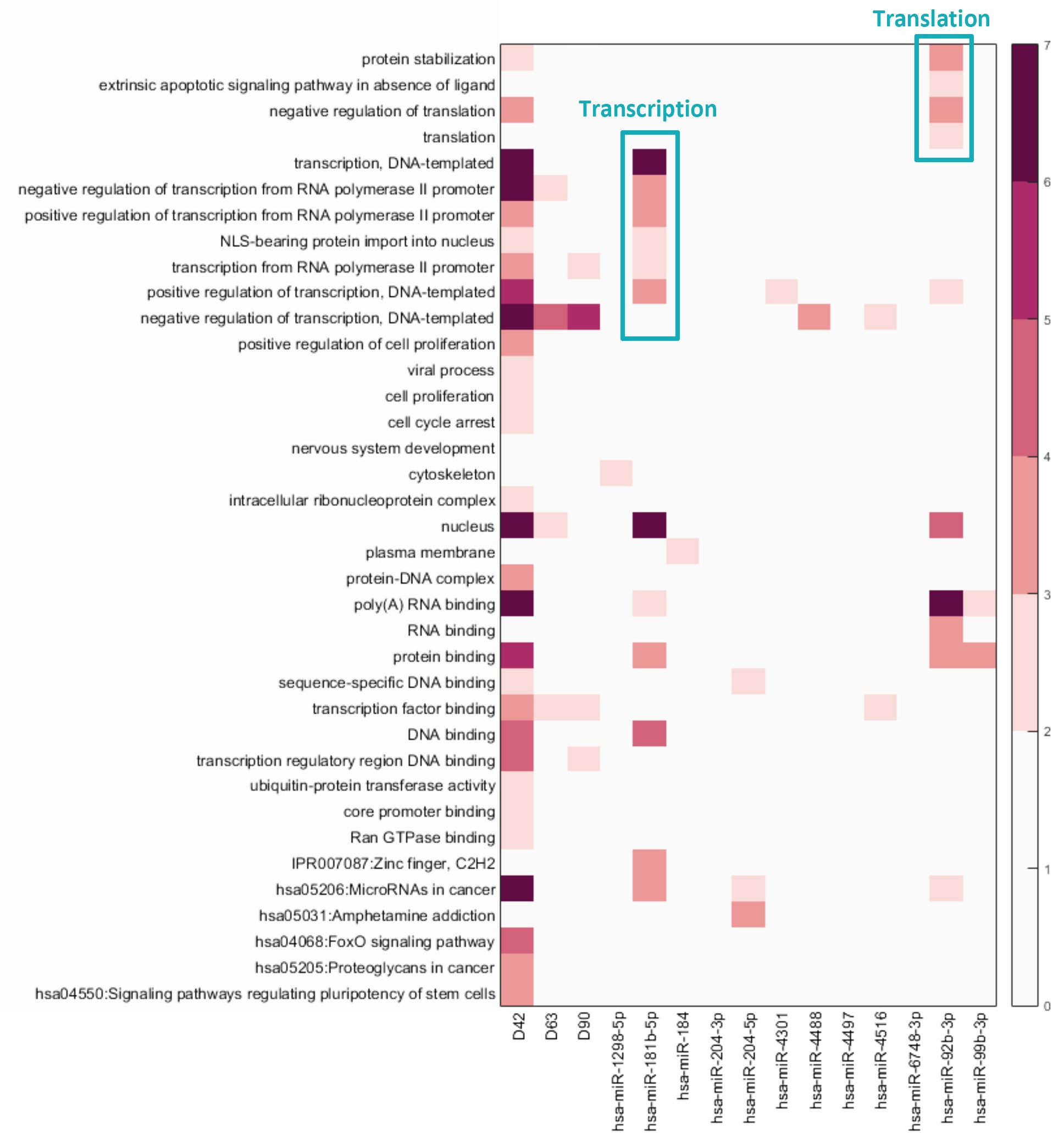


**Supplemental Figure 5 (SF5). Gene Ontology and pathway enrichment analysis.** The heat map shows the P-value of enriched GO categories for each of the identified miRNAs at D42, D63 and D90. GOs from unbiased analysis show significant biological processes associated with predicted target genes. When analyzed by developmental time point, GO categories exhibited significantly higher enrichment at D42, for transcription, RNA binding, protein binding, positive regulation of cell proliferation and nuclear transcription factor binding. When analyzed by individual miRNAs, some showed significant enrichment for GO terms associated to specific cellular mechanisms. Specifically, hsa-miRNA-181b-5p targets showed significant enrichment in mechanisms related to transcription, including DNA-templated transcription and transcription from RNA polymerase II promoter, while hsa-miRNA-926-3p targets showed significant enrichment in translation related to protein stabilization and negative regulation of translation. P values were determined using Fisher’s exact test. The color scale represents significance in white (low, p-value ≥ 10−2), light orange (medium, 10−4 < p-value < 10−2), and red (high, p-value ≤ 10−4).


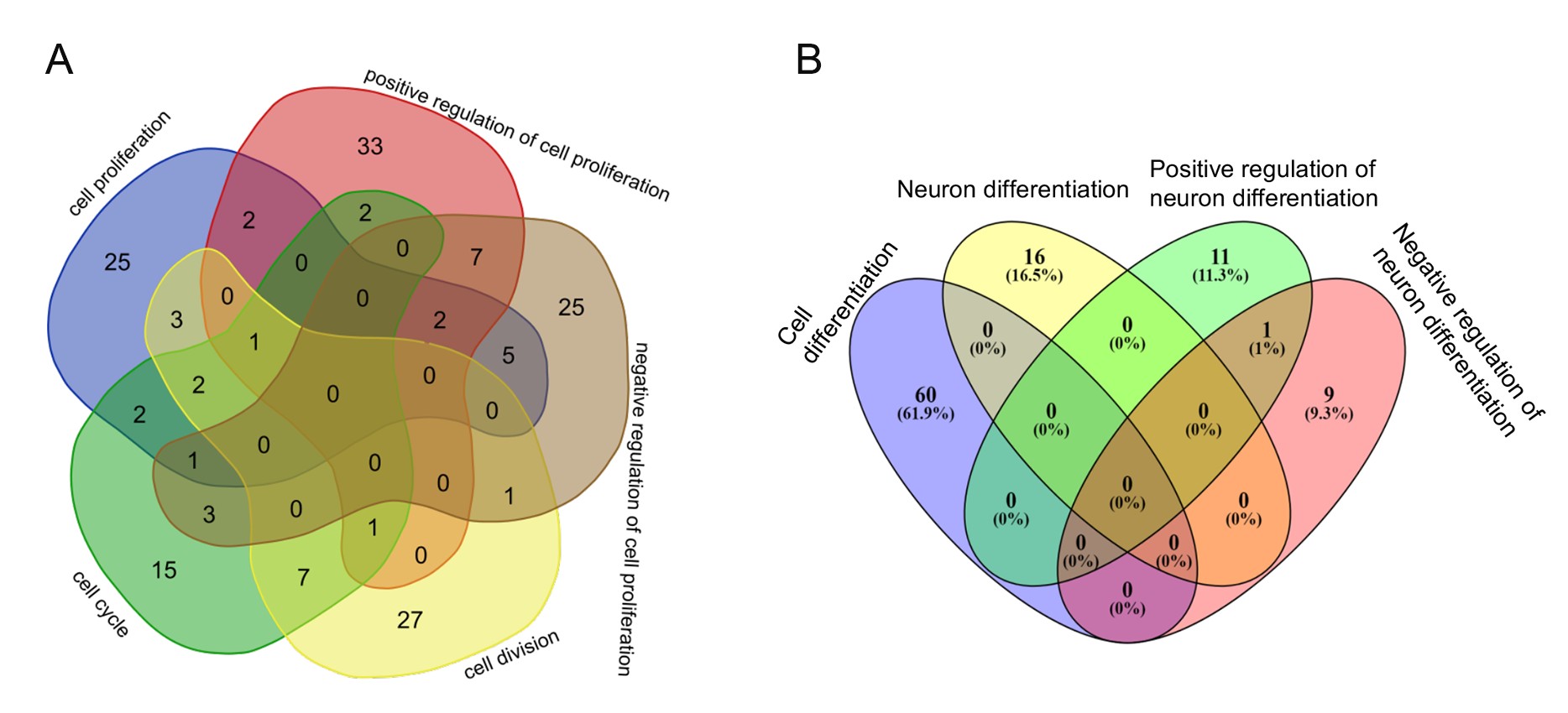


**Supplemental Figure 6 (SF6). Correlation of GO terms and predicted target genes**. A) Venn diagram illustrating the number of predicted target genes corresponding to GO terms associated to cell proliferation (“cell proliferation” vs “positive regulation of cell proliferation” vs “negative regulation of cell proliferation” vs “cell cycle” vs “cell division”). B) Venn diagram illustrating the number of predicted target genes corresponding to GO terms associated to cell differentiation (“cell differentiation” vs “neuron differentiation” vs “positive regulation of neuron differentiation” vs “negative regulation of neuron differentiation.

| **Gene** | **Analysis** | **Control hRPCs** | **EV-treated hRPCs** |
| --- | --- | --- | --- |
| ILDR2 | RNA Seq | 31.54 | 27.71 |
|  | qPCR | 1.00 | 0.77 |
| CCSER2 | RNA Seq | 844.46 | 722.02 |
|  | qPCR | 1.00 | 0.88 |
| FAM117B | RNA Seq | 152.43 | 114.43 |
|  | qPCR | 1.00 | 0.84 |
| PVRL1 | RNA Seq | 762.09 | 757.03 |
|  | qPCR | 1.00 | 0.95 |
| CTDSPL | RNA Seq | 972.88 | 985.32 |
|  | qPCR | 1.00 | 1.17 |

**Supplemental Table ST3. hRPC co-cultured with EVs released from hiPSC-derived 3D retinal organoids showed downregulation of predicted targeted genes by both RNASeq and qPCR.** EV miRNA targeted genes in hRPCs showed similar patterns of expression between RNAseq and qPCR in control (untreated) and hiPSC-derived 3D retinal organoid EV treated samples. Four of the five selected genes showed a trend of downregulated expression in EV-treated samples compared to controls, while

1 gene (CTDSPL) had an opposite pattern. The average of two to three replicates for each sample was used for each target gene, with the expression level in control set as 1.0.

| **gene** | **baseMean** | **log2FoldChange** | **lfcSE** | **stat** | **pvalue** |
| --- | --- | --- | --- | --- | --- |
| AC002467.7 | 16.15594168 | 1.556574858 | 0.647974375 | 2.402216692 | 0.016296052 |
| AC005229.7 | 12.99837769 | 1.420355996 | 0.717441091 | 1.979752781 | 0.047731315 |
| AC068282.3 | 23.92450058 | 1.753467227 | 0.567804943 | 3.08815069 | 0.002014063 |
| AL662800.1 | 41.56535494 | 1.308904619 | 0.47512314 | 2.754874492 | 0.005871465 |
| APOC1 | 29.93660814 | 1.078979931 | 0.49034978 | 2.200429111 | 0.027776464 |
| CCDC79 | 2.542130585 | 4.798118116 | 2.041637289 | 2.350132485 | 0.01876673 |
| CD14 | 21.02321876 | 1.50923931 | 0.587362063 | 2.569521262 | 0.010183914 |
| CTD-2078B5.2 | 14.57729594 | 1.372512364 | 0.668661133 | 2.052627701 | 0.040108699 |
| CTD-2308G16.1 | 1.995610908 | 4.425732803 | 2.184783184 | 2.025708013 | 0.042794715 |
| CXCL10 | 5.259138481 | 3.263665692 | 1.409554781 | 2.315387621 | 0.020591728 |
| DDX11L2 | 17.53129044 | 1.248377265 | 0.629239931 | 1.983944762 | 0.047261998 |
| FABP5 | 598.5973082 | 1.034178269 | 0.272545337 | 3.794518305 | 0.00014793 |
| FAM71E1 | 24.33832001 | 1.549325347 | 0.547089884 | 2.831939309 | 0.004626663 |
| FLI1-AS1 | 31.03228944 | 1.197428715 | 0.521254661 | 2.29720481 | 0.021607089 |
| FOLR3 | 3.265516819 | 3.631713496 | 1.844639426 | 1.968793166 | 0.048976847 |
| HIST1H2AG | 1531.610928 | 1.107331159 | 0.277643355 | 3.988322204 | 6.65E-05 |
| HIST1H2BC | 2557.215776 | 1.113342575 | 0.241834926 | 4.603729463 | 4.15E-06 |
| HIST1H2BN | 1739.203818 | 1.059446849 | 0.25767292 | 4.111595617 | 3.93E-05 |
| HIST2H2BB | 26.05291151 | 1.042146122 | 0.526835146 | 1.978125663 | 0.047914532 |
| HIST2H2BE | 3765.568665 | 1.052392001 | 0.264412221 | 3.980118611 | 6.89E-05 |
| MIR125B1 | 51.39706369 | 1.168924507 | 0.404526023 | 2.889615109 | 0.003857138 |
| MIR193B | 10.34157378 | 1.96445373 | 0.83749201 | 2.345638773 | 0.018994506 |
| NCBP2-AS1 | 1.870609698 | 4.349004593 | 2.164107553 | 2.009606495 | 0.044472853 |
| NTS | 488.3408378 | 1.472957901 | 0.255698858 | 5.760518108 | 8.39E-09 |
| PTMAP4 | 60.12171714 | 1.127845182 | 0.536637994 | 2.101687162 | 0.035580689 |
| RBP7 | 4.230990914 | 5.542273763 | 1.819599018 | 3.045876432 | 0.002320032 |
| RLBP1 | 30.8057436 | 1.144834463 | 0.493031527 | 2.322030944 | 0.020231269 |
| RNU1-32P | 30.3531729 | 1.288820798 | 0.620007615 | 2.078717692 | 0.037643306 |
| RNU1-60P | 22.45280862 | 1.560436808 | 0.621979065 | 2.508825288 | 0.012113338 |
| RNU2-15P | 8.169232859 | 2.025379744 | 1.000099079 | 2.025179092 | 0.042848978 |
| RP11-126K1.6 | 18.28777527 | 1.331001477 | 0.631861902 | 2.106475281 | 0.035163088 |
| RP11-18B16.2 | 2.16257366 | 4.553641003 | 2.128638773 | 2.13922675 | 0.032417309 |
| RP11-234P3.4 | 6.785816054 | 3.126130567 | 1.203431887 | 2.597679685 | 0.0093856 |
| RP11-430B1.2 | 7.261300178 | 2.087851615 | 1.010115764 | 2.066942908 | 0.038739534 |
| RP11-455F5.3 | 12.28676062 | 1.778456881 | 0.762583294 | 2.332147709 | 0.019692922 |
| RP11-467J12.4 | 5.596727536 | 2.316627687 | 1.157868817 | 2.000768699 | 0.045417322 |
| RP11-545E17.3 | 26.89686762 | 1.252305395 | 0.521299973 | 2.402274044 | 0.016293497 |
| RP11-831F12.4 | 21.01446027 | 1.40673728 | 0.627031819 | 2.243486277 | 0.024865474 |
| RPL11P5 | 23.74050979 | 1.43925124 | 0.565759894 | 2.543925888 | 0.010961434 |
| RPL41 | 3163.682761 | 1.12049213 | 0.236039025 | 4.747063039 | 2.06E-06 |
| TOMM22P5 | 22.08509837 | 1.321964156 | 0.556185188 | 2.376841716 | 0.017461579 |
| UBAC2-AS1 | 18.75622413 | 1.208749362 | 0.598579572 | 2.019362869 | 0.043449518 |

**Supplemental Table ST4 Table of all genes upregulated 2-fold change, 0.05 p-value in hiPSC- derived 3D retinal organoid EV treated hRPCs.**
